# Evaluation of a novel SARS‐CoV‐2 rapid antigenic test diagnostic value in respiratory samples; is the reported test accuracy similar to values in the real‐world? A cross‐sectional study

**DOI:** 10.1002/hsr2.765

**Published:** 2022-08-10

**Authors:** Hossein Hatami, AhmadReza Rezaeian

**Affiliations:** ^1^ Department of Public Health and Safety School of Public Health and Environmental and Occupational Hazard Control Research Center, Shahid Beheshti University of Medical Sciences Tehran Iran; ^2^ Faculty of Medicine Shahid Beheshti University of Medical Sciences Tehran Iran; ^3^ Urology Research Center Tehran University of Medical Sciences Tehran Iran

**Keywords:** antigen test, COVID‐19 symptoms, diagnostic Value, RT‐PCR, SARS‐CoV‐2

## Abstract

**Background and Aims:**

Although reverse transcription‐polymerase chain reaction (RT‐PCR) assay was introduced as the gold standard to detect SARS‐CoV‐2, the method was known to be time‐consuming besides the requirement for an equipped laboratory. This survey aims to investigate a novel SARS‐CoV‐2 antigen test as a diagnostic tool in COVID‐19 patients to overcome these limitations in addition to evaluating COVID‐19 population characteristics.

**Methods:**

A retrospective cross‐sectional study was carried out during the first semester of 2021, and about 1070 nasopharyngeal samples were collected to compare the E‐Health Barakat Company SARS‐CoV‐2 antigen rapid test results with RT‐PCR reports as the reference method.

**Results:**

Totally 537 participants were included in this study for employing RT‐PCR and the antigen test sequentially. The novel antigen rapid test sensitivity is considered 21.09% in the real world, though 81% in the manufacturer's instruction has been mentioned. Moreover, the most revealed manifestations were found respiratory symptoms and fatigue sensations.

**Conclusion:**

This study is the first one on evaluating the SARS‐CoV‐2 antigen test in our country. Although the novel antigen assay was found quick and easy to perform, the test performance was very disappointing. The extensive false‐negative results made it an inappropriate candidate for mass screening.

## 1 INTRODUCTION

Coronaviruses are well‐studied RNA viruses in the Coronaviridae family, order Nidovirales, containing positive single‐stranded RNA, which is identified as the most enormous viral RNA genome.^1^, ^2^ The virus is surrounded by an envelope with crown‐like spikes on its surface, resulting in a spherical shape roughly 80–120 nm in diameter.^3^

The first coronavirus and later the first human coronavirus were detected in the 1930s and 1960s.^4^, ^5^ Almost all coronaviruses are responsible for self‐limit and mild diseases in humans.^2^ Surprisingly, in the ongoing century, three new zoonotic coronaviruses have been discovered, resulting in severe illnesses and global crises.^2^, ^6^ In 2002, severe acute respiratory syndrome (SARS) was first diagnosed in China, and a decade later, in 2012, the Middle East respiratory syndrome (MERS) was discovered in Saudi Arabia. Nearly 750 and 850 deaths occurred due to these novel coronaviruses sequentially.^2^ Lastly, severe acute respiratory syndrome coronavirus 2 (SARS‐CoV‐2) was identified in China, giving rise to the coronavirus disease‐2019 (COVID‐19) outbreak necessarily. It was March 11, 2020 when the World Health Organization (WHO) introduced this disease as a pandemic.^2^, ^7^ The term pandemic is defined as a universal emergence of a disease in one or more areas.^3^ Thus, unfortunately, this recent novel coronavirus could lead to much more burdens than other forms. According to the WHO website, as of November 26, 2021, over 259,500,000 confirmed SARS‐CoV‐2 infections are reported worldwide and consequently COVID‐19 has claimed the lives of about 5,000,000 people globally, in addition to remarkable social and economic problems.^3^, ^8^, ^9^

Respiratory droplets are mostly in charge of COVID‐19 transmission when the person's distance is less than 1 m with an infected case.^3^, ^4^ Therefore, COVID‐19 spreads quickly, and the novel coronavirus could be found easily in more than 180 countries, including our country, Iran.^1^, ^4^, ^10^

In accordance with the current studies, various signs and symptoms are introduced for the infection, including respiratory and extrarespiratory manifestations such as cough, fever, malaise, diarrhea, headache, dermatological signs, anosmia, and ageusia, and so forth.^6^, ^11^, ^12^, ^13^

Although, as mentioned before, many complaints are known to be related to the SARS‐CoV‐2 infection, a great proportion of individuals declare nonspecific manifestations or are left asymptomatic, causing considerable challenges and demand for expanding testing to manage virus outbreaks.^7^, ^14^, ^15^

Fundamentally, the deoxyribonucleic acid (DNA) sequencing assays can be employed to investigate undetermined mutations; even so, these technologies are laborious and costly. Hence, reverse transcription‐polymerase chain reaction (RT‐PCR) methods have been developed to recognize SARS‐CoV‐2.^16^ RT‐PCR for COVID‐19 diagnosis was presented in January 2020 and to date is considered as the recommended technique and the gold standard for SARS‐CoV‐2 detection in respiratory samples by means of nasopharyngeal and oropharyngeal swabs.^17^, ^18^, ^19^ Even though RT‐PCR is a standard diagnostic assay, the process lasts approximately 4 h to reveal the result, in addition to the need for expert personnel and special supplies presently limited.^14^, ^20^, ^21^ To improve this vital requirement, alternative methods such as rapid antigen tests were implemented which detect SARS‐CoV‐2‐specific antigen in an individual's samples commonly obtained from the patient's nasal cavity.^18^, ^19^

Antigen tests are low‐cost and time‐saving assays that release results in less than half an hour. Administering these assays does not require skilled technicians and a specific equipped laboratory. Owing to these advantages, antigen tests could be applied for mass screening in the population.^18^, ^19^

Our survey was carried out to evaluate a novel SARS‐CoV‐2 antigen test widely used in our country, Iran.

## 2 MATERIAL AND METHOD

A retrospective cross‐sectional study was undertaken during the first 6 months of 2021, between January 20, 2021 and July 22, 2021, to investigate E‐Health Barakat Company SARS‐CoV‐2 Antigen Rapid Test as a diagnostic tool for patients with COVID‐19 in the population visited at Shemiranat Medical Center.

The survey was accepted by the Ethics Review Committee of Shahid Beheshti University of Medical Sciences (IR.SBMU.PHNS.REC.1400.076) since the verbal informed consents were received from all the patients clearly to employ their files for research and publication purposes. Also, the physician appointments and tests were done totally free.

Individuals referred to the center were all Iranian and could be from all over the country, though most of them lived in Tehran, the capital city of Iran. Participants admitted to the medical center were asked about COVID‐19 symptoms, duration, contact, and demographic information. Physicians in the clinic visited patients and those with probable SARS‐CoV‐2 infection reporting COVID‐19 symptoms or an epidemiological risk factor were included in the study.

Elucidated in previous research, during the first week of the COVID‐19 disease, specifically on Day 5, the viral load is at the highest level and as a consequence it is introduced at the best time for employing antigenic tests.^2^, ^22^ Testing during this period can lead to antigenic tests to be more sensitive and, in developing countries, such kinds of strategies can definitely help to overcome limited resources. Accordingly, in this study participants were all visited on Day 5 of illness, based on patients' first COVID‐19‐related complaints, for applying RT‐PCR and the antigen rapid test consecutively.

**Figure 1:**
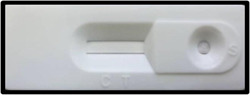
E‐Health Barakat Company SARS‐CoV‐2 antigen rapid test cassette before adding the sample

The E‐Health Barakat Company SARS‐CoV‐2 Antigen Rapid Test kit includes a specific buffer (the manufacturer did not disclose its composition), nasopharyngeal swab, tube, and cassette (Figure 1). The datasheet notified there were no cross‐reactions with analytes, including albumin, bilirubin, hemoglobin, glucose, and uric acid. Furthermore, other microbes like MERS, influenza B virus, influenza A virus subtype H1N1 and H3N2, Group A and B Streptococcus, Adenovirus type 1 and 2, respiratory syncytial virus type A and B, human coronavirus NL63, 229E, and OC43 cannot interfere the reaction or make any changes in the result.

Trained staff studied the instructions supplied and used swabs to obtain nasopharyngeal samples from each participant in a specific ventilated and isolated room to decrease the risk of transmission. Skilled personnel with personal protective equipment tilted the patient's head back and inserted the swabs through a nostril to reach the nasopharynx. The nasopharyngeal swabs should be rotated both sides on their own axis 5 s independently. Later, the swabs with specimens were placed in a tube containing the buffer and stirred effectively 10 times. Lastly, the swab will be removed and stored as protocol in a biological safety receptacle until getting collected. Then three drops of the taken sample were put on the determined specimen site on the cassette marked with the patient's data.

**Figure 2:**
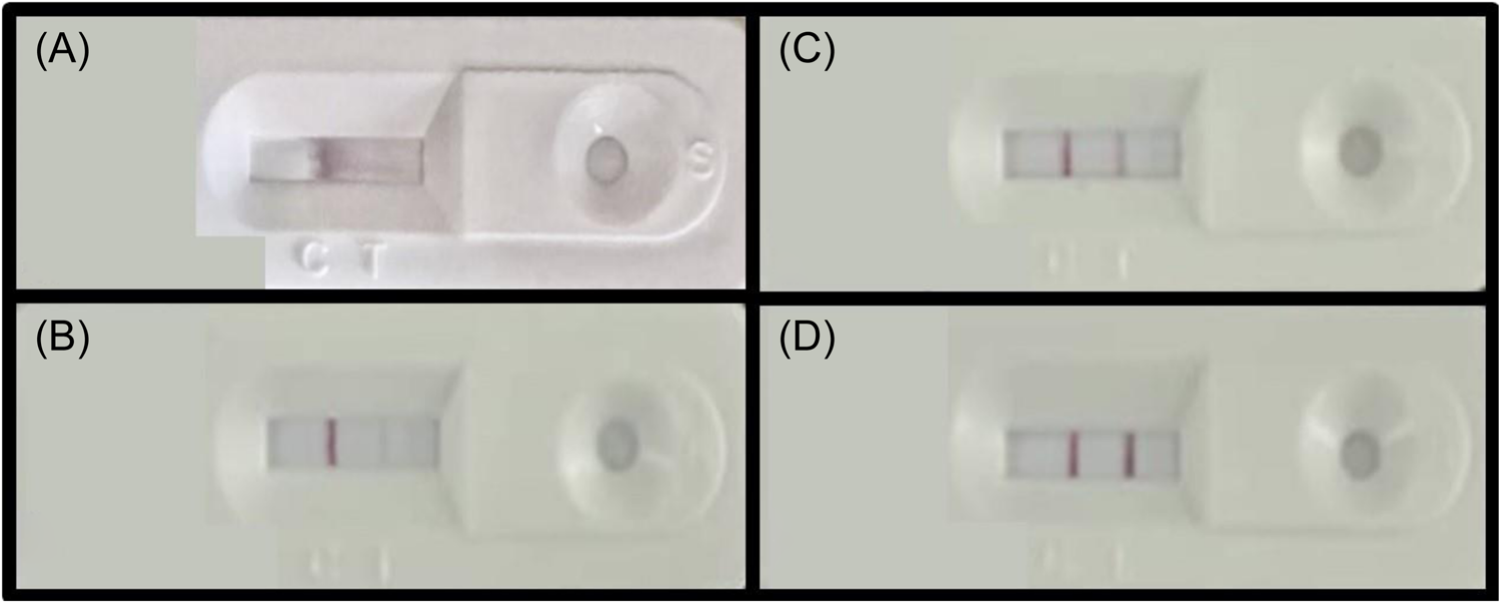
Examples for antigen rapid test interpretation (patient's information is covered). Presentation of (A) an antigenic test cassette processing the result, (B) an antigenic test cassette interpreted as a negative result, (C) an antigenic test cassette interpreted as a positive result with low antigenic load, (D)an antigenic test cassette interpreted as a positive result.

After 15–20 min, the result can be interpreted. Once one red line with a C (control) mark is seen, it means that the device cannot detect SARS‐CoV‐2 antigen in the sample but the examination was done correctly. Results without a remarkable control line were not acceptable even if the T (test) line colored was lonely. Finally, if two colored lines were emerged simultaneously, the individual was introduced as a positive case, as shown in Figure 2.

Moreover, another specificnasopharyngeal swab was applied at the same time for running the RT‐PCR assay for diagnostic purposes. The procedure was generally similar but the samples were obtained from the other nostril to collect enough epithelial cells. Subsequently, the samples in a particular package were transported to an equipped laboratory and reports were collected on the same day as the reference method. The samples with cycle thresholds (CT) less than 40 were interpreted as positive results. Any deviation in any part of the whole process made the case excluded from our investigation.

Confirmed cases with positive RT‐PCR reports were known candidates for follow‐up, so they got informed incessantly and were asked for home quarantine. Besides, to be cautious, suspected cases with negative reports were explained about the test errors.

Eventually, for data statistical analysis, SPSS Version 20 was used. Mean (SD, 95% confidence interval [CI]) and percentage (numerator, denominator) for normally distributed quantitative variables and qualitative variables were employed respectively. The two‐sided Pearson *χ* ^2^ test was applied for data statistical comparison and *p* values less than 0.05 were defined as statistically significant results.

## 3 RESULTS

A total of 537 individuals were included and at least 1074 nasopharyngeal samples were collected. The employed gold standard RT‐PCR methods and antigen rapid tests reported 128 and 32 positive results, respectively, with no missed test results (data are shown in Table 1).

**Table 1:** True positive, false negative, false positive, and true positive data are demonstrated here

	RT‐PCR test	
Antigen test	Positive	Negative
Positive	27	5
Negative	101	404

Abbreviation: RT‐PCR, reverse transcription polymerase chain reaction.

Overall, all the tested cases were Iranian and the mean (SD, 95% CI) age of the study population was 46.93 (15.25, 45.62–48.24) years old. Among the selected cases, 52.7% (283/537) were male, while this value changed to 53.1% (68/128) in the definite COVID‐19 population.

**Figure 3:**
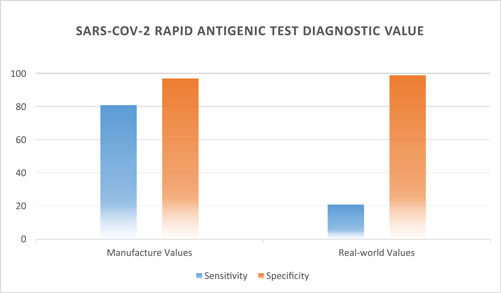
The chart demonstrates diagnostic values reported in the manufacturer's instruction versus in the real world. The real‐world sensitivity value is found to be approximately one‐fourth of the mentioned value in the manufacturer's datasheet.

Our main goal was analyzing the performance characteristics of E‐Health Barakat Company SARS‐CoV‐2 Antigen Rapid Test. Based on gold standard RT‐PCR assay results, the mentioned test showed its overall sensitivity 21.09% (27/128), specificity 98.78% (404/409), positive predictive value 84.38% (27/32), negative predictive value 80% (404/505), likelihood ratio positive 17.25% (0.210/0.012), likelihood ratio negative 0.8% (0.789/0.987), and the infection prevalence evaluated 23.84% (128/538). Albeit the manufacturer's datasheet described a study done with about 60 definite SARS‐CoV‐2 samples and final calculated sensitivity was 81% besides specificity being considered at 97% (Figure 3).

Furthermore, our survey provided some characteristics of COVID‐19 patients. Most of these laboratory‐confirmed participants, 79.68% (102 patients among 128 cases), were married. In total, 8.59% (11 out of 128), 11.71% (15 out of 128), 29.68% (68 out of 128), and 50% (64 out of 128) of individuals outlined their degree as doctorate, master, bachelor, and lower than a bachelor's degree respectively.

Body mass index (BMI) is calculated for SARS‐CoV‐2‐positive cases showing 18.11% (23/127) obese, 43.3% (55/127) overweight, 35.43% (45/127) normal weight, and 3.14% (4/127) underweight persons. In other words, this population mean (SD, 95% CI) BMI was 26.41 (4.61, 25.60–27.22) kg/m^2^.

We also assessed the symptoms of the infected population. Manifestations including upper and lower respiratory, gastrointestinal, dermatological, anosmia and ageusia, fever, fatigue, and headache are questioned. Almost half of the COVID‐19‐confirmed cases revealed respiratory syndromes (64/128) and fatigue sensation (58/128). The next most reported manifestation was fever, which is seen in approximately one‐third of patients (42/128). The only significant correlation with positive RT‐PCR reports was found for upper respiratory symptoms such as coughing (*p* = 0.002) evaluated by the Pearson *χ* ^2^ test (two sided). More accurately, *p* values of positive RT‐PCR correlation and individuals' symptoms including lower respiratory, gastrointestinal, dermatological, anosmia and ageusia, fever, fatigue and headache were calculated 0.14, 0.64, 0.32, 0.15, 0.09, 0.94, and 0.28 by two‐sided Pearson *χ* ^2^ test, respectively.

## 4 DISCUSSION

To the best of our knowledge, this study is the first one on the evaluation of the SARS‐CoV‐2 antigen rapid test in the Iranian population.

Since COVID‐19 was introduced as the most lethal pandemic of the new century and compared with fatal and terrifying outbreaks like the Spanish flu in 1918 and the HIV/AIDS pandemic in the current century, we have to consider this disease seriously and related measures should be undertaken as soon as possible.^2^

Although RT‐PCR assays are determined as a molecular test consisting of a series of gene amplification, antigen tests are known as a set of immunoassays, a subgroup of biochemical methods. The antigenic test mechanism of identification is generally dependent on the specific and suitable antibody which is selected to have a high affinity for binding with the target molecule. Therefore, this choosing process is still one of the great challenges in this method and the test sensitivity could be affected by using monoclonal antibodies.^17^, ^23^

The other problem in these assays is the concentration of the biomarkers. Compared to the nucleic acid amplification technique, this method does not include an amplifying step for an indicator and that is why its sensitivity is predominantly low. Thus, the viral load can impact the results and, accordingly, we did all the sampling at the highest viral stage.^23^

As a consequence, however, the antigen rapid tests are assumed to be very handy and functional, but their validity and real‐world performance should be recognized.

Our findings demonstrated dramatically less sensitivity (21.09% vs. 81%) and higher specificity (98.78% vs. 97%) than the values mentioned in the supplied instructions. By reason of obeying all the manufacturer's recommendations perfectly, the only accused factor for this difference could be the samples quality, though we obtained the best sample possible in the real world.^24^

Using only the specimens with the highest viral load reported in the literature, as we asked our patients to get tested on Day 5 of illness, excluding the effect of cycle thresholds distribution in our samples.^9^, ^22^, ^25^

Inevitably, the prominent limitation of the rapid antigen test is the large number of false negatives, which makes the kit improper for mass screening. Newly published works identified higher sensitivity compared with our applied kit, while this assay sensitivity is still determined to be inferior to the RT‐PCR method.^14^, ^20^, ^24^, ^25^

Optimistically, the antigen assay new generations can overcome its limitations and the health institutions use these potent tests to help the overwhelmed virology laboratories in the current pandemic.^22^

Our results for COVID‐19 infection presentations are on the contrary to previous findings in Lebanon, USA, Taiwan, and UK which claimed fever as the most reported symptom, showed fatigue sensation and respiratory symptoms as the most common ones.^2^, ^4^, ^11^, ^13^ Even though our confirmed COVID‐19 population declared fatigue sensation mainly, just about one‐third complained about fever symptom (44.9% vs. 33.1%). Align well with our data, other surveys disclosed the fatigue sensation as COVID‐19 symptom repeatedly.^4^, ^13^ The data about respiratory symptoms including cough are consistent with numerous studies outlining this manifestation at a high rate.^2^, ^4^, ^11^, ^13^

Furthermore, male gender, old age, and obesity are known as risk factors making cases prone to severe stages of COVID‐19 infection.^9^ We found out that more than half of our patients were male, similar to a study showing females were less affected by SARS‐CoV‐2.^1^ The last mentioned risk factor is the reason for the necessity of calculating BMI in all COVID‐19 cases as suggested in a paper from Indonesia.^26^ Greater than 650,000,000 adults and 124,000,000 children and adolescents are struggling with the obesity pandemic globally in the twenty‐first century.^27^ Obesity increases the person's vulnerability to SARS‐CoV‐2 infection in addition to progression to critical phases.^27^, ^28^ Hence, individuals with this independent risk factor and other comorbidities should be prioritized in COVID‐19 management and testing.^28^, ^29^ About one‐fifth of our cases suffered from obesity, which is given more vigilance.

Ultimately, our findings elucidated that almost 80% of our participants were married. This report should increase social concerns about family problems since a partner's physical illness is recognized as a remarkable stressor which may lead to martial breakdown and other consequences. For instance, after World War two, as a stressor, marital dissatisfaction and divorce rates grew.^30^

We acknowledge the limitations of our survey. The research data are limited to the first semester of 2021 and the COVID‐19 outbreak, in addition to Iranian ethnicity. There is no information about other probable conditions.

## 5 CONCLUSION

Taken together, E‐Health Barakat Company SARS‐CoV‐2 Antigen Rapid Test sensitivity is determined to be low and disappointing, though the assay was fast and easy to perform. Consequently, the antigen assays are likely to miss lots of COVID‐19 cases, causing virus transmission to be trouble free. This huge false‐negative population is the reason for not recommending the use of this antigen test for COVID‐19 screening particularly in Iranian ethnicity.

## AUTHOR CONTRIBUTIONS

Both authors contributed to conceptualization, methodology, and validation. AhmadReza Rezaeian is the principal investigator who provided, cured, analyzed and visualized the data. Moreover, AhmadReza Rezaeian administrated the project and wrote the original draft. Hossein Hatami carefully supervised the research and also reviewed and edited the manuscript. All authors have read and approved the final version of the manuscript.

## CONFLICT OF INTEREST

The authors declare no conflict of interest.

## TRANSPARENCY STATEMENT

AhmadReza Rezaeian affirms that this manuscript is an honest, accurate, and transparent account of the study being reported; that no important aspects of the study have been omitted; and that any discrepancies from the study as planned (and, if relevant, registered) have been explained.

## Data Availability

AhmadReza Rezaeian had full access to all of the data in this study and takes complete responsibility for the integrity of the data and the accuracy of the data analysis. The data that support the findings of this study are available from the corresponding author upon reasonable request.
